# A metabolomic approach to understand the solid-state fermentation of okara using *Bacillus subtilis* WX-17 for enhanced nutritional profile

**DOI:** 10.1186/s13568-019-0786-5

**Published:** 2019-05-04

**Authors:** Wai Kit Mok, Yong Xing Tan, Jaslyn Lee, Jaejung Kim, Wei Ning Chen

**Affiliations:** 1 School of Chemical and Biomedical Engineering, Nanyang Technological University, 62 Nanyang Drive, N1.2-B1-35, Singapore, 637459 Singapore; 2 Interdisciplinary Graduate School, Nanyang Technological University, 50 Nanyang Avenue, Singapore, 639798 Singapore; 3 Advanced Environmental Biotechnology Centre, Nanyang Environment and Water Research Institute, Nanyang Technological University, 1 CleanTech Loop, 1 CleanTech One #06-08, Singapore, 637141 Singapore

**Keywords:** *Bacillus subtilis*, Okara, Fermentation, Metabolomics, Food waste valorisation

## Abstract

Okara is a major agro-waste produced from the soybean industry. To hydrolyze the okara and enable nutrient release, a strategy to valorize okara using solid-state fermentation with food grade *Bacillus subtilis* (*B*. *subtilis*) WX-17 was carried out. The study showed that fermentation of okara with *B*. *subtilis* WX-17 improved its overall nutritional content. The total amino acids content increased from 3.04 ± 0.14 mg/g in unfermented okara to 5.41 ± 1.21 mg/g in okara fermented with *B*. *subtilis* WX-17. Total fatty acids content increased from 153.04 ± 5.10 to 166.78 ± 2.41 mg/g okara, after fermentation. Antioxidant content (DPPH) also increased by 6.4 times after fermentation. To gain insight into the mechanism, gas chromatography–mass spectrometry analysis was carried out. In total, 49 metabolites were detected, which could be classified mainly into carbohydrates, TCA cycle metabolites, amino acids and fatty acids. The decrease in carbohydrate metabolites, showed that glycolysis was upregulated. This would have provided the energy and metabolic flux towards the amino acid and fatty acid pathway. This is also in line with the increased amino acids and fatty acid production seen in okara fermented with *B*. *subtilis* WX-17. The findings of this work demonstrated the potential of using *B. subtilis* WX-17 fermentation, to enhance the nutritional profile of okara. This could serve as a potential low-cost animal feed or incorporated into the human diet.

## Introduction

Okara, also known as soy pulp is a major agro-waste produced from the soybean industry which produces soymilk and bean curd. Approximately 1.1 kg of okara are produced from 1 kg of soybean (O’Toole 1999). Okara is highly nutritious. It contains approximately 50% fiber, 25% protein, 10% lipid as well as a myriad of other high-value compounds such as isoflavones, coumestans, saponins, phytosterols, lignins and phytates. These compounds have been shown to exhibit numerous physiological and therapeutic functions such as the prevention of cardiovascular diseases (CVD) in humans. However, a large amount of okara are being disposed of in landfills and incineration plants annually due to their unpalatable and insoluble nature (Li et al. 2012b). It is estimated that around 14 million tonnes of okara are generated worldwide annually. The countries include Japan, Korea, China and Singapore, which contribute 800,000 tonnes, 310,000 tonnes, 2.8 million tonnes and 11,000 tonnes, respectively (Li et al. 2012b; Seong et al. 2015).

To utilize the highly nutritive compounds in the okara, pre-treatment is required to release the nutrients from the insoluble okara. Previous studies have shown that enzymes secreted by microorganisms during fermentation can hydrolyse complex macromolecules such as fatty acids, proteins and fibres into smaller and more soluble nutrients. It also reduces the amount of antinutritional factors present in okara including trypsin, phytic acid, lectin and tannin (Paredes-Lopez and Harry 1989). Various microorganisms such as *Aspergillus sp., Aspergillus niger* and *Aspergillus ficuum* had been studied for their ability to produce phytases which inhibited the antinutritional factor phytate. This had been shown to reduce the bioavailability of calcium, zinc and iron (Pandey et al. 2001; Schlemmer et al. 2009).

Solid state fermentation (SSF) is the culture of microorganism using solid substrate in the absence of liquid to produce desirable products. SSF has shown to be effective in enhancing the nutritional content of a complex substrate. This is because microorganism secretes abundant enzymes which catabolizes complex macromolecules into simpler forms. For example, a recent study conducted by Dessie et al. showed that fruit and vegetable wastes that underwent solid state fermentation using *Aspergillus niger* and *Rhizopus oryzae* increased in succinic acid which is an important metabolite in the tricarboxylic acid (TCA) cycle and have numerous health benefits such as antioxidant properties and strengthening of the immune system (Dessie et al. 2018; Saif and Fumio Hashinaga 2005). Bacteria, yeast and fungi are commonly used in SSF. *Rhizopus*, *Lactobacillus, Streptococcus, Aspergillus and Bacillus* are some of the most common microorganism used in solid state fermentation of food material (Hesseltine 1987).

In recent years, SSF have received increasing amount of attention from researchers and industrial players since several studies performed on colourings, flavourings, additives and other desirable products for the food industry had shown that SSF can achieve higher yield compared to SLF (Rodriguez-Couto and Sanromán 2006). It is considerably cheaper and more environmentally friendly as compared to the more commonly used submerged liquid fermentation (SLF). Some of the advantages of SSF over SLF include reduced probability of contamination due to lack of moisture and simple media composition since most nutrients are in the solid substrate. SSF also allows the use of simple reactor design due to the concentrated nature of solid substrate (Mienda and Idi 2011).

*Bacillus subtilis* (*B. subtilis*) is a microorganism of interest for fermentation of okara, due to its ability to secrete enzymes, which can break down the macromolecules in okara, as well as the ability to increase antioxidant activity. The objective of this study was to investigate the effects of SSF on okara using a strain of food grade *B. subtilis* WX-17, which was isolated in this study, from *Natto*. Then, an untargeted metabolomic approach using gas chromatography-mass spectrophotometry (GC–MS) was carried out to analyse the value-added products produced in okara fermented with *B. subtilis* WX-17. The mapping of metabolites unto the metabolomic pathways would also provide an important insight into the mechanisms behind solid-state fermentation of okara with *B. subtilis* WX-17. Till date, based on our knowledge, there exist a gap in the utilization of bacteria in valorising okara from a metabolomic perspective. The findings of this study could open up the possibility of using fermented okara as a low-cost animal feed or even supplement the human diet which could go a long way towards alleviating the global food security issue.

## Materials and methods

### Chemicals

Glycerol, nutrient broth, methanol, ribitol, methoxamine hydrochloride, N-methyl-N-(trimethylsilyl) trifluoroacetamide (MSTFA), trimethylchlorosilane (TMCS), sodium chloride, acetic acid, heptadecanoic acid, ethanol, chloroform, BF3-methanol, hexane, γ-aminobutyric acid, dimethylformamide (DMF), 1-1,-diphenyl-2-picryl-hydrazil (DPPH).

### Microorganism

*Bacillus subtilis* WX-17 was isolated from Marumiya Kyushu Ichiban Natto. This strain has been deposited in NCIMB with the accession number NCIMB 15204. The isolation was carried out by adding 20 mL of sterile water to 3 natto beans in a falcon tube and vortexed for 5 min to extract the microorganism. The cell suspension was serial diluted and plated onto nutrient agar plates and incubated at 37 °C for 24 h. A single colony was inoculated into 5 mL of nutrient broth and incubated at 37 °C for 24 h and subsequently stored in aliquots containing 50% glycerol at − 80 °C.

### Bacterium identification

A single colony was inoculated in 5 mL of nutrient broth and incubated overnight. The bacterial DNA were then isolated using Bio Basic EZ-10 Spin Column Fungal Genomic DNA Mini-Prep Kit. Next, PCR was carried out to amplify the 16S rDNA gene of the bacteria using the forward and reverser primer 27F (5′ AGA GTT TGA TCM TGG CTC AG 3′) and 1492R (5′ GGT TAC CTT GTT ACG ACT T 3′), respectively. PCR was performed with the following parameters: 35 cycles at 98 °C 10 s for denaturation, 55 °C 5 s for annealing, 72 °C 2 min for elongation and 68 °C 10 min for extension followed by cooling to 4 °C. Gel electrophoresis was then carried out on the PCR sample to remove other contaminants and the DNA purified using QIAquick Gel Extraction Kit (250) before sequencing. 16s rRNA sequencing (Genbank accession number MK559744) was outsourced to Bio Basic Asia Pacific Pte Ltd using Sanger dideoxy sequencing technology. The obtained 16s rRNA sequence (GenBank accession number MK559744) was then compared with other 16s rRNA sequences using the BLAST algorithm (https://blast.ncbi.nlm.nih.gov/Blast.cgi).

### Source of okara

Fresh okara samples were kindly provided by Vitasoy International Singapore Pte Ltd, Singapore. Okara were separated into aliquots and sealed in air tight polyethylene bags and stored at − 20 °C in the dark.

### Fermentation

*Bacillus subtilis* WX-17 was inoculated into 5 mL of nutrient broth and incubated at 37 °C for 24 h which served as the stock culture. 10 g of okara was inoculated with *B. subtilis* WX-17 at a concentration of 10^6^ CFU/g of okara in a petri dish. The petri dish was then covered with 2 layers of cling film. The first layer was pressed onto the inoculated okara and the second layer was wrapped across the surface of the petri dish. Both layers were punctured with numerous holes using a sterile pin to maintain aeration and moisture content within the petri dish and subsequently fermentation was carried out at 37 °C for 72 h. A beaker of water was placed in the incubator to maintain the moisture content. The fermented okara were then freeze-dried and stored at − 20 °C until further analysis.

### Sample preparation for metabolomic, fatty and amino acids analyses

For the metabolomic analysis, 3 mL of methanol were added to 900 mg of fermented okara and raw okara (control), respectively. The samples were then homogenized using Fastprep-24™ 5G Homogenizer. Homogenizing was carried out for 30 s at 5 min interval for 5 times. The tubes were placed in a box of ice after each homogenizing cycle to cool the samples. Next, the samples were centrifuged at 9000*g* for 10 min at 4 °C. The supernatant was extracted and filtered through a 0.22 µm filter. 10 µL of ribitol (2 mg/mL) were added into 1.5 mL of filtered supernatant as the internal standard (IS). Samples were then vortexed for 30 s and allowed to dry in a heat block at 30 °C, overnight. Then, 100 µL of methoxamine hydrochloride (20 mg/mL pyridine) was added to the lyophilized samples for methoximation to protect the carbonyls and incubated at 37 °C for 60 min. Next, silylation was carried out by adding 200 µL of *N*-methyl-*N*-(trimethylsilyl) trifluoroacetamide (MSTFA) with 1% trimethylchlorosilane (TMCS) and subsequently incubated at 70 °C for 30 min. The samples were then centrifuged for 30 min at 15,330*g* and 150 µL of supernatant were transferred to glass vials and sent for GC–MS analysis.

For fatty acids analysis, 10 mg of fermented okara and 10 mg of fresh okara (control) were weighed and placed into eppendorf tubes. 1000 µL of 0.9% NaCl solution and 200 µL of acetic acid were then added. 10 µL of 10 mg/mL heptadecanoic acid dissolved in ethanol was added to the extraction solvent to serve as IS. The solvents were then homogenized as described above. Then, 3 mL of a chloroform–methanol 2:1 mixture was added, and the samples were inverted several times, vortexed vigorously for 5 min, and centrifuged at 10,000*g* for 10 min at 4 °C. The chloroform layer (bottom, 1 mL) was collected and dried overnight at 30 °C. The dried lipid residue was re-dissolved in 500 µL BF3-methanol 10% (FLUKA, 15716) and incubated in a sealed vial in a 95 °C heater for 20 min. FAMEs were extracted with 300 µL *n*-hexane after the addition of 300 µL saturated NaCl in water. Samples were vortexed for 5 min and centrifuged at 14,800 rpm for 5 min. 150 µL of sample (top layer) was transferred into glass vials for GC–MS analysis.

For amino acids analysis, 4 mg of fermented okara and 4 mg of raw okara (control) were resuspended in 200 µL of 6 M HCl. 20 µL of γ-aminobutyric acid (10 mg/mL) were added as IS. The tubes were sealed and baked for 24 h in an oven at 105 °C. The cell hydrolysate was dried at 95 °C in a heat block. After drying, 20 µL of DMF and 20 µL of MSTFA were added. The tubes were sealed and incubated at 85 °C for 1 h. Samples were then centrifuged at 14,800 rpm for 5 min and supernatant were transferred to glass vials. 40 µL of DMF were added into the glass vials and the vials were inverted a few times before sending for GC–MS analysis.

### GC–MS method for metabolomic, fatty and amino acids analyses

Metabolomic analysis including carbohydrates and TCA cycle metabolites were carried out via GC–MS. The GC–MS system (Agilent Technologies 7890A-5975C) was equipped with a HP-5MS, 5% Phenyl-Methyl-Silox capillary column (30 m × 0.250 mm id.; 0.25 μm film thickness; Agilent J&W Scientific, Folsom, CA, USA). 1 µL of samples were injected into the system by the autosampler in splitless mode. The injector temperature and ion source temperature were set at 250 °C and 230 °C, respectively. The oven temperature was as follows: 75 °C for 4 min, ramped to 280 °C at the rate of 4 °C/min, and held at 280 °C for 2 min. Data were acquired in full scan mode from 35 to 600 m/z with a 0.3 s of scan time. Metabolites were identified using the NIST08 mass spectral library based on mass spectral similarity. Samples were normalized with respect to the IS, ribitol, before comparison.

For fatty acid analysis, the injector temperature and ion source temperature were set at 250 °C and 230 °C, respectively. The oven temperature was as follows: 80 °C for 1 min, ramped to 250 °C at the rate of 7 °C/min, and held at 250 °C for 8 min. Data were acquired in full scan mode from 50 to 600 m/z at 2.66 scans per second. Metabolites were identified using the NIST08 mass spectral library based on mass spectral similarity. Samples were normalized with respect to the IS, heptadecanoic acid before comparison.

For amino acid analysis, solvent delay was set at 2 min 30 s. The injector temperature and ion source temperature were set at 250 °C and 230 °C, respectively. The oven temperature was as follows: 160 °C for 1 min, ramped to 290 °C at the rate of 20 °C/min, ramped again to 310 at 20 °C/min and held at 310 °C for 1 min. Data were acquired in full scan mode from 180 to 550 m/z at 3.85 scans per second. Metabolites were identified using the NIST08 mass spectral library based on mass spectral similarity. Samples were normalized with respect to the IS, γ-aminobutyric acid before comparison.

### Antioxidants analysis

300 µL of ethanol was added to 100 mg of sample and homogenized as described above. The samples were then centrifuged at 10,000 rpm for 5 min. 150 µL of the supernatant were transferred to new tubes and added with 100 µL of 1,1,-diphenyl-2-picryl-hydrazil (DPPH) solution and 250 µL of ethanol. The samples were then incubated in a dark place for 30 min at room temperature. The absorbance of the mixture was measured at 515 nm with ethanol as blank using Nanodrop 2000c Spectrophotometer. The activities of the samples were evaluated with respect to trolox equivalent- % signal inhibition calibration curve whereby % signal inhibition is defined as: $\% \; Signal \; Inhibition = \left( {1 - \frac{{A_{s} }}{{A_{o} }}} \right) \times 100.$

A_s_ is defined as the absorbance of the samples and A_o_ is defined as the absorbance of pure DPPH.

### Statistical analysis

All experiments were conducted in triplicates. Statistical analysis was carried out using MetaboAnalyst 4.0 (Xia et al. 2011, 2012; Xia and Wishart 2016). Data scaling was carried out using mean-centering and divided by the standard deviation of each variable prior to partial least squares discriminant analysis (PLS-DA) and heatmap analysis. The heatmap was also constructed using Euclidean distance measurement and ward clustering algorithm.

### Nucleotide sequence accession number

The 16s rRNA sequence of WX-17 was deposited in the GenBank database with the accession number MK559744.

## Results

### Metabolic profiles of fermented and unfermented okara

**Fig. 1:**
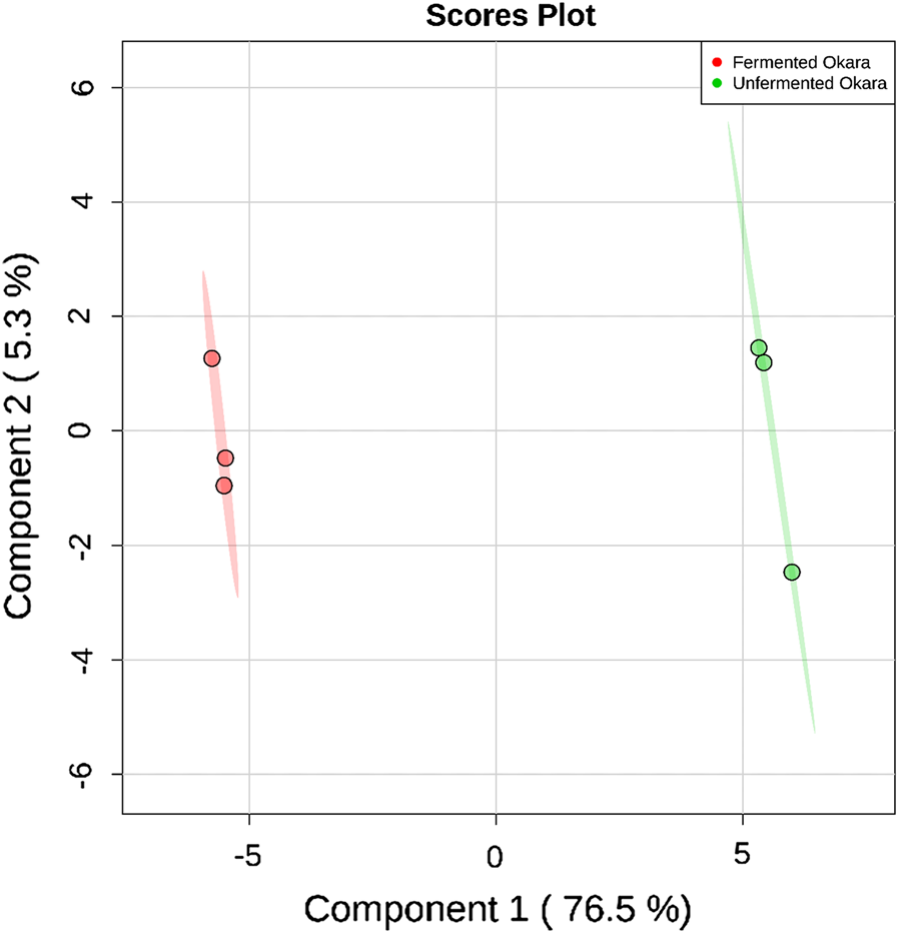
PLS-DA score plot of all metabolites found for fermented and unfermented okara. The green and red highlights denoted the 95% confidence region. Explained variance are shown in brackets

A metabolomics analysis using GC–MS provided an overview on the metabolic profiles between the fermented and unfermented okara. Statistical analysis (PLS-DA and heatmap) was carried out to understand the changes between the samples. These changes observed in the level of each metabolite, helped to shed light on the effects of *B. subtilis* WX-17 fermentation on okara. In total, 49 metabolites were detected. Figure 1 showed the PLS-DA score plot of the fermented and unfermented okara. The green and red highlights denoted the 95% confidence region. The first principal component accounted for 76.5% of the total variance while the second principal component accounted for 5.3% of the total variance which combined to explain a total of 81.8% of the variance. This showed that the first component largely explained most of the variance between the samples. From the PLS-DA score plot (R^2^ = 99.8% and Q^2^ = 98.3%), clear and distinct separations between the unfermented okara and the fermented okara along the first principal component were observed. This showed that during fermentation, the metabolic profile of okara had changed significantly.

**Fig. 2:**
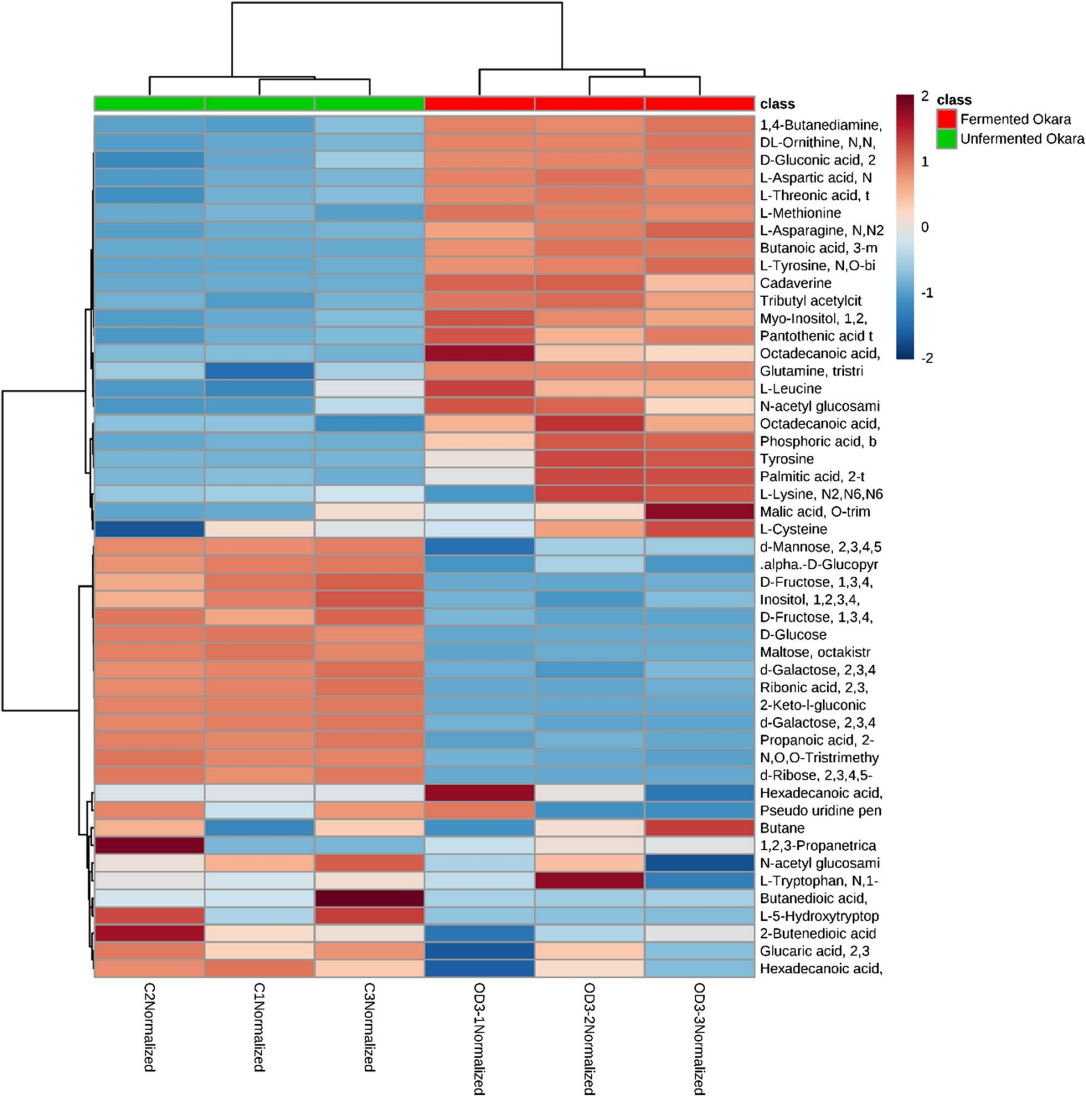
Heatmap analysis correlating the metabolites of fermented and unfermented okara

Although the PLS-DA score plot provided a visual representation of the difference in the metabolic profile between fermented and unfermented okara, it did not provide details about the specific metabolites. Hence a clustering heatmap was constructed to provide a more visual breakdown of the metabolites that changed after fermentation (Fig. 2). From the heatmap, unfermented okara had high amount of carbohydrates such as fructose, ribose, glucose, galactose, mannose and maltose. In comparison, the levels of carbohydrates were lower in fermented okara. In addition, fermented okara had higher amounts of amino and fatty acids as compared to unfermented okara. This suggested that *B. subtilis* WX-17 consumed the carbohydrates in okara for its growth and might have produced proteases and lipases to break down proteins and lipids in okara, into simpler amino acids and fatty acids (Lesuisse et al. 1993; Yang et al. 2000).

### Carbohydrates and TCA cycle metabolites

**Fig. 3:**
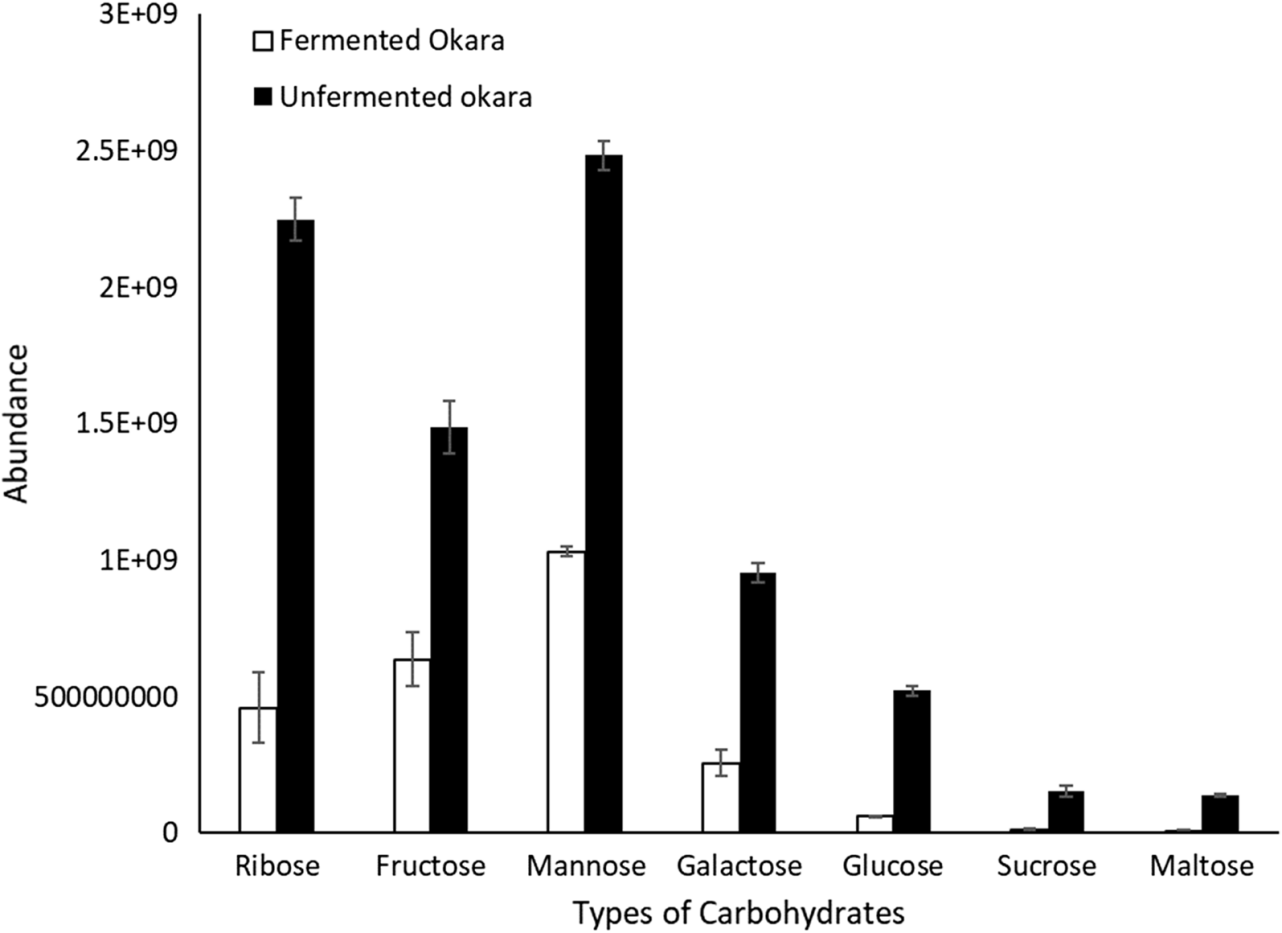
Abundance of all the carbohydrates detected during analysis

**Fig. 4:**
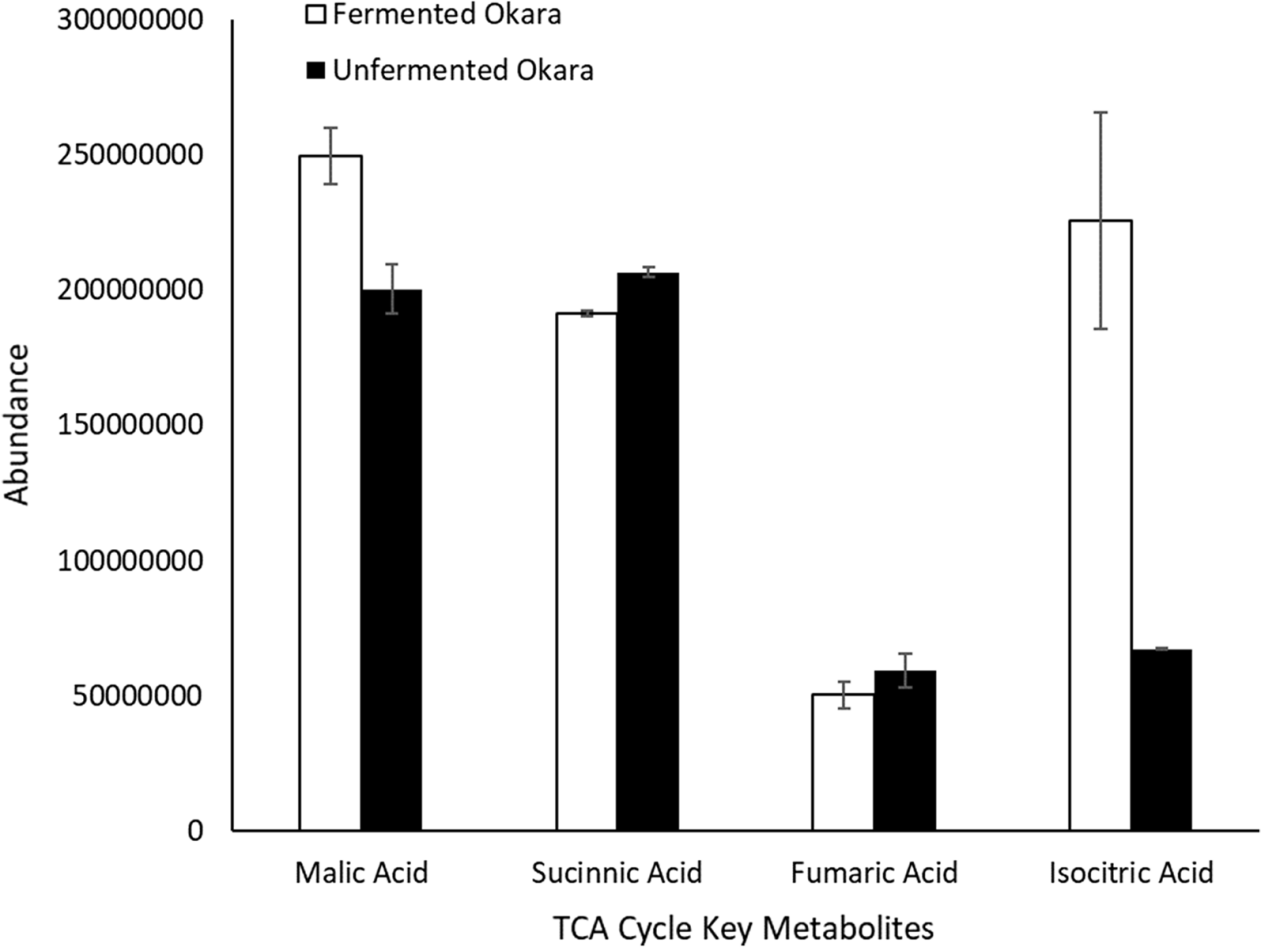
Abundance of all the TCA cycle key metabolites detected

To better illustrate the effects of fermentation on fermented and unfermented okara, the results for carbohydrate metabolites and metabolites involved in the TCA cycle were presented in Figs. 3 and 4 respectively. In total, 7 types of carbohydrates were detected. They were ribose, fructose, mannose, galactose, glucose, sucrose, and maltose. This result agreed with previous findings by Li et al. and Hou et al. which showed that the polysaccharides in soybeans and okara were ribose, galactose, glucose, fructose, sucrose, mannose and other minor components such as verbascose, pinitol and myo-inositol (Hou et al. 2009; Li et al. 2012a). The abundance of all the different types of carbohydrates were reduced after fermentation (Fig. 3).

From the metabolomics analysis, it was shown that isocitric acid and malic acid increased while succinic acid and fumaric acid decreased (Fig. 4). Most notably, isocitric acid increased by approximately 3 times. Recent studies have postulated on the potential benefits of isocitric acid. For example, Omar et al. suggested that isocitric acid contains antioxidant properties that can help to combat oxidative stress of the brain and liver in mice by decreasing the brain lipid peroxidation and inflammation, liver damage as well as DNA fragmentation (Abdel-Salam et al. 2014). Another study suggested that isocitric acid can help in combating hypoxia or hypoxic conditions such as fatigue, dizziness to more serious conditions such as hypercapnia and organ failure as it is the only metabolite in the TCA cycle that can unblock succinate dehydrogenate which would promote cell respiration even under stressful environment (Kamzolova et al. 2018).

### Amino acids and fatty acids metabolites

From the heat map, it was shown that amino acids and fatty acids levels increased after fermentation. To reaffirm these findings, the amount of amino acids and fatty acids before and after fermentation were determined using GCMS protocols which were specific for amino and fatty acids. These specific protocols are more sensitive towards their respective target metabolites. Table 1 showed the absolute value of amino acids (mg/g okara) in fermented and unfermented okara. The results in Table 1 showed that all amino acids increased after fermentation with the total amount increasing by almost two-fold after fermentation. Notably, the essential amino acids leucine, phenylalanine and glutamic acid increased the most at 2.26, 2.42 and 2.12 times, respectively.

**Table 1:** Changes in amino acids in absolute value (mg/g dried okara) for fermented and unfermented okara

mg/g okara	Control (raw okara)	Fermented okara
Glycine	0.183 ± 0.0441	0.329 ± 0.104
Valine	0.0228 ± 0.00291	0.0458 ± 0.00457
Proline	1.28 ± 0.442	2.15 ± 0.591
Leucine	0.303 ± 0.0684	0.685 ± 0.175
Serine	0.130 ± 0.0309	0.141 ± 0.0188
Threonine	0.138 ± 0.0391	0.151 ± 0.00157
Phenylalanine	0.0799 ± 0.0306	0.194 ± 0.0179
Aspartic acid	0.200 ± 0.0703	0.292 ± 0.0278
Glutamic acid	0.611 ± 0.0211	1.30 ± 0.182
Lysine	0.0694 ± 0.00989	0.0856 ± 0.0156
Tyrosine	0.0235 ± 0.00215	0.0439 ± 0.00352
Total amino acids	3.04 ± 0.136	5.41 ± 1.21

Results are as *mean ± standard deviation (3 replicates)*

Fatty acids specific analysis suggested that there was negligible change in stearic and palmitic acids levels. However, linoleic and oleic acids levels were shown to increase by 2.93 and 2.37 times, respectively (Table 2).

**Table 2:** Changes in fatty acids in absolute value (mg/g dried okara) for fermented and unfermented okara

mg/g okara	Control (raw okara)	Fermented okara
Stearic acid	60.3 ± 2.00	55.0 ± 5.24
Oleic acid	3.39 ± 1.02	8.04 ± 2.87
Linoleic acid	9.61 ± 3.31	28.2 ± 9.55
Palmitic acid	79.8 ± 1.93	75.6 ± 5.37
Total fatty acids	153.04 ± 5.09	166.78 ± 2.41

Results are as *mean ± standard deviation (3 replicates)*

### Antioxidant activity

**Fig. 5:**
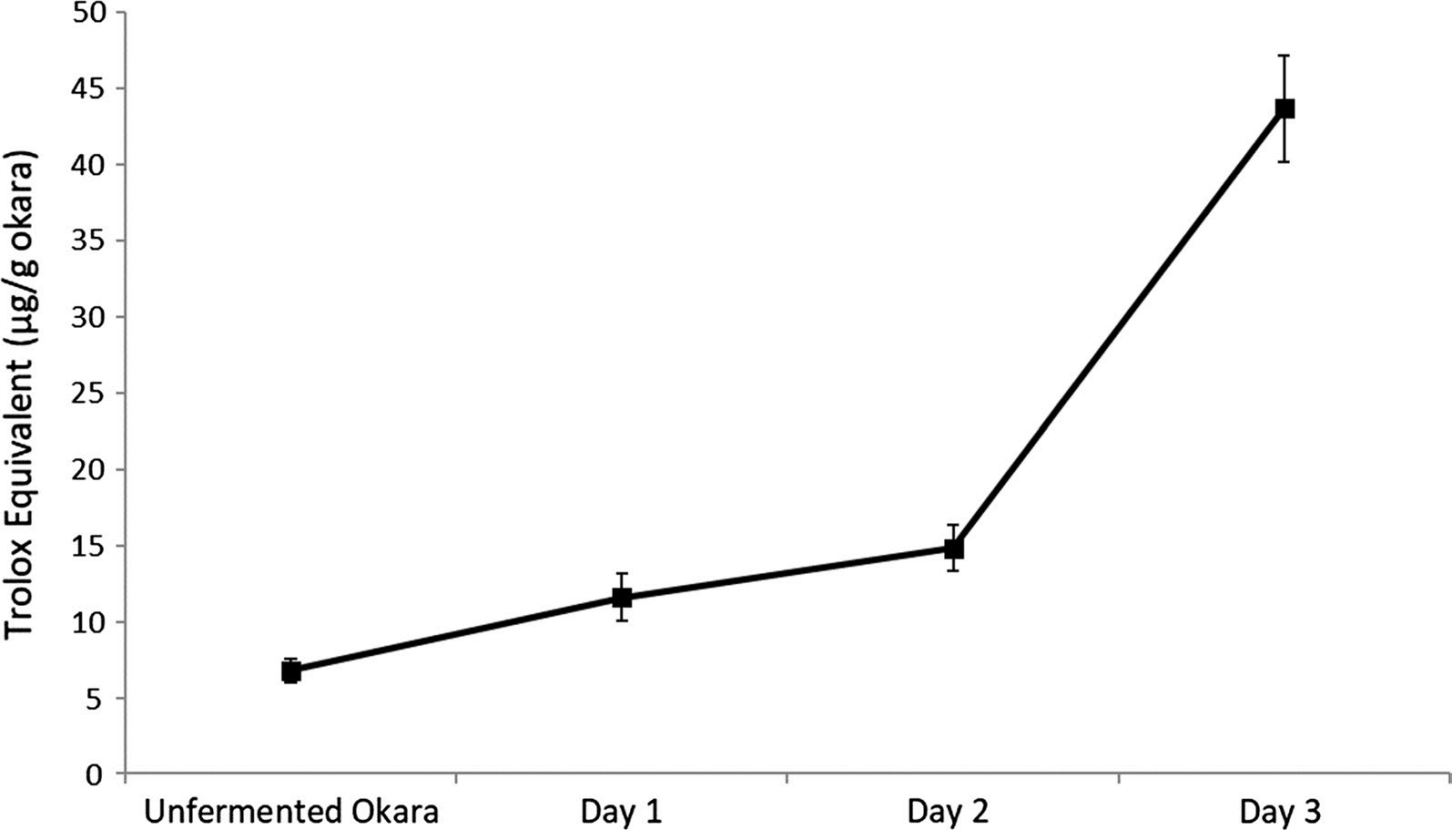
DPPH scavenging activity of fermented and unfermented okara across 72 h expressed in terms of Trolox equivalent (µg/g okara)

The DPPH radical scavenging activity of fermented okara was shown to have increased during fermentation (Fig. 5). Fermented okara showed the highest DPPH radical scavenging activity at 43.68 µg Trolox equivalent/g dried okara after 72 h of fermentation. As compared to the DPPH radical scavenging activity of unfermented okara at 6.79 µg Trolox equivalent/g dried okara, fermented okara displayed an increased in DPPH radical scavenging activity by approximately 6.4 times.

## Discussion

Metabolomics analysis showed that the overall metabolite profile of okara changed after *B. subtilis* WX-17 fermentation (Figs. 1 and 2). Most of the carbohydrate metabolites decreased after fermentation. This indicated that it was being consumed by *B. subtilis* WX-17 which could utilize the carbohydrates through glycolysis, to produce increased amounts of the energy molecule acetyl-CoA. This precursor could then enter the amino acids and fatty acid pathway. This suggested that the microorganism was able to utilise the carbon source present within okara and use it for metabolism to produce other components. It had been suggested that glucose is the preferred carbon source for *B. subtilis* (Singh et al. 2008; Tian et al. 2015). In this study, analysis of the various sugar pathways suggested that most other forms of carbohydrate were converted into glucose before being used for other processes.

The increase in amino acids were confirmed as shown in Table 1. This could be due to *B. subtilis* producing extracellular proteases which would breakdown the proteins in okara into amino acids thereby contributing to their increase after fermentation. The increase in amino acids is important as they can provide as a rich source of nitrogen that are essential to living organisms. For example, yeast such as *Rhodosporidium toruloides* and *Saccharomyces cerevisiae* requires nitrogen for the synthesis of amino acids, proteins, DNA and RNA (Cruz et al. 2002; Evans et al. 1984).

Fatty acid level also increased after okara fermentation (Table 2). Particularly, the increase in both linoleic acid and oleic acid levels, were desirable as studies have reported various health benefits when these fatty acids were consumed. Linoleic acid is a polyunsaturated fatty acid that contains numerous purported health benefits such as anti-obesity, anti-carcinogenesis, anti-atherosclerosis, anti-diabetic, osteosynthetic and immunomodulation effects (Benjamin and Spener 2009; Nagao and Yanagita 2005). Likewise, oleic acid is a monounsaturated fatty acid that had been linked to a reduction in coronary heart disease due to its ability to reduce LDL-cholesterol, thrombogenicity, LDL-oxidative susceptibility as well as insulin sensitivity factors (Lopez-Huertas 2010). The increase in linoleic acid and oleic acid were expected as *B. subtilis* are known to produce lipases that catalyses the hydrolysis of fatty acids (Ma et al. 2006; Sánchez et al. 2002).

Endogenous metabolic processes or exogenous chemicals in food system may generate free radicals which may cause oxidative damages by oxidizing biomolecules resulting in tissue damage or even cell death. Numerous traditional fermented food such as miso, tempeh, sufu and douche have free radical scavenging ability (Zhu et al. 2008). Therefore, it is of interest to analyse if *B. subtilis* WX-17 fermented okara displayed the same ability. In this regard, analysis showed that antioxidant activity increased by 6.4 times which strengthened the case of fermented okara as a functional food for animals.

**Fig. 6:**
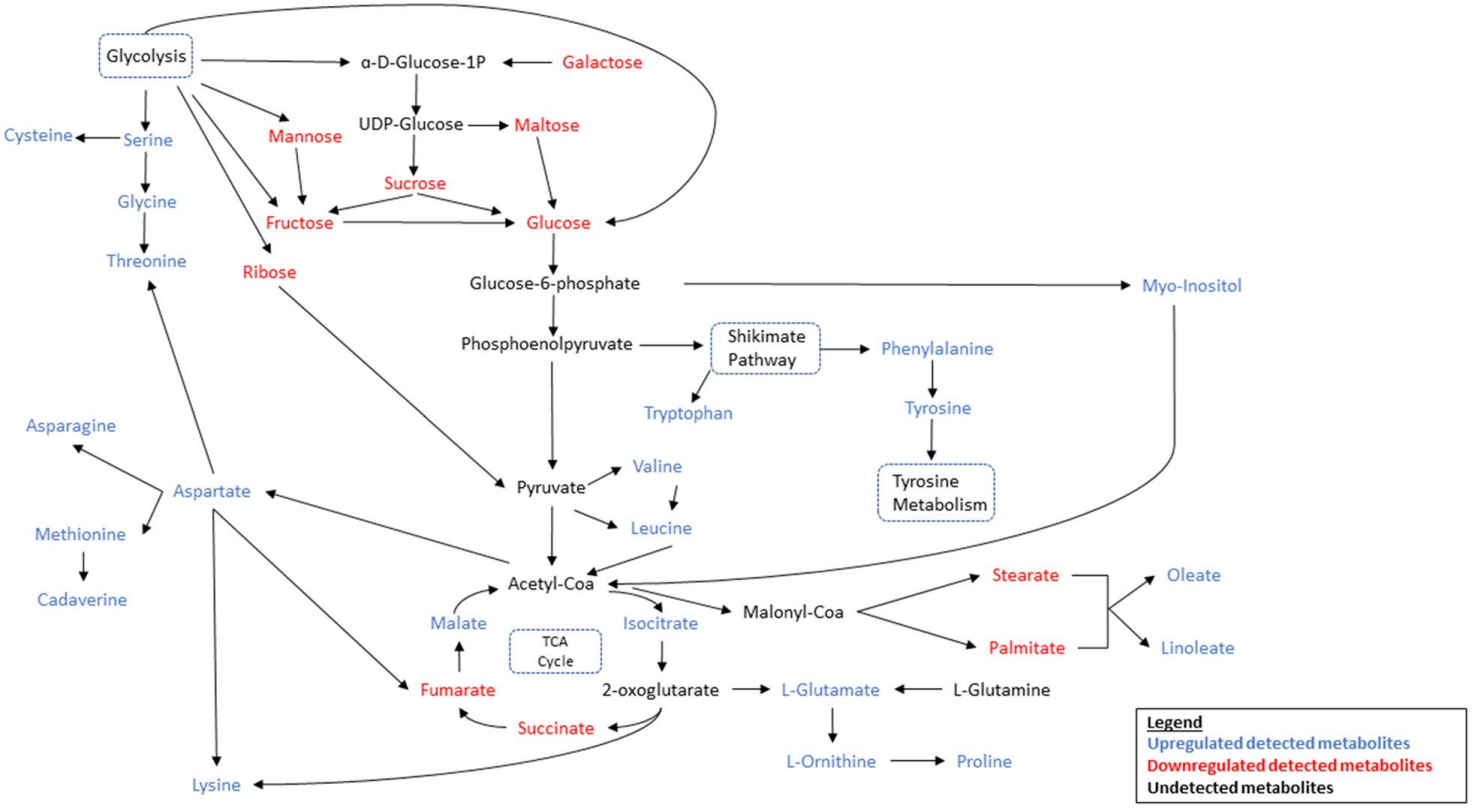
Metabolic pathway analysis of all detected carbohydrates, amino acids and fatty acids after fermentation of okara

To better understand the metabolic flux during fermentation, a metabolic pathway analysis was performed that would allow a hypothetical insight into the various metabolic pathways which were up regulated or down regulated during fermentation (Fig. 6). The pathway analyses were performed with reference to the Kyoto Encyclopaedia of Genes and Genomes (KEGG) database (Kanehisa et al. 2017; Kanehisa and Goto 2000; Kanehisa et al. 2016; Ogata et al. 1999).

The precursor for glycolysis is glucose. It is converted into pyruvate through the intermediate glucose 6-phosphate (G6P) and phosphoenolpyruvate (PEP). The analysis showed that apart from ribose, all other forms of carbohydrates can be converted into glucose. Mannose can be converted into fructose through the enzyme mannose isomerase in a reversible reaction. Fructose is in turn converted into glucose via xylose isomerase. Both mannose and fructose were also produced through glycolysis through the intermediates mannan and d-fructose-2-phosphate respectively. The overall decrease in both mannose and fructose suggested that the rate of consumption is greater than the rate of production.

Glycolysis also produced sucrose through the intermediate UDP-glucose and sucrose synthase. Sucrose can then be converted into fructose and glucose through sucrose alpha-glucosidase. UDP-glucose can also be converted to maltose via maltodextrin which were then converted to glucose through maltose phosphorylase. Galactose can be converted into UDP-glucose via UDP glucose pyrophosphorylase.

The KEGG database suggested no direct pathway for conversion of ribose to glucose. This suggested that the reduction in ribose after fermentation is due to direct consumption of ribose by *B. subtilis* WX-17. This was supported by studies which showed that *B. subtilis* exhibited increased sporulation when provided with a mixture of glucose and ribose as carbon source compared to a medium with glucose as the sole carbon source (Warriner and Waites 1999).

One of the main intermediates of glycolysis, G6P can be converted into myo-inositol, a carboxylic sugar through myo-inositol 1-phosphatase (inositol phosphate metabolism pathway). During fermentation, *B. subtilis* WX-17 releases phytase which hydrolyses phytic acid, an antinutrient present in okara to produce myo-inositol which might explain the increased amount of myo-inositol detected after fermentation (Chen et al. 2015; Kerovuo et al. 1998). Myo-inositol can then be converted into acetyl-coA through malonate-semialdehyde dehydrogenase which can then enter the TCA cycle (inositol phosphate metabolism pathway).

Another intermediate in glycolysis, phosphoenolpyruvate (PEP) was involved in the shikimate pathway that produce tryptophan through a reversible reaction with (3-indole)-glycerol phosphate. The increased level of tryptophan detected suggested that the forward reaction (3-indole-glycerol phosphate to tryptophan) occurred at a higher rate than the backward reaction. Both phenylalanine and tyrosine are involved in the shikimate pathway as well and can be interconverted through prephenate which is an intermediate in the shikimate pathway. Their increased levels after fermentation suggested that reactions along the pathway are skewed towards phenylalanine and tyrosine metabolism rather than quinate.

Both valine and leucine which are essential amino acids were produced from the glycolysis intermediate, pyruvate (valine, leucine, isoleucine biosynthesis pathway). Valine is produced from the intermediate, 2-oxoisovalerate which can also be irreversibly converted into leucine. In addition, pyruvate can also be converted into leucine through pyruvate metabolism. Increased levels of valine and leucine suggested that their rate of synthesis is greater than their rate of degradation as leucine can be broken down into acetyl-coA. This implied that the bulk of the acetyl-coA were converted from pyruvate rather than leucine.

TCA cycle is a chain of reactions that are used by aerobic organisms to release stored energy in acetyl-coA through oxidation into adenosine triphosphate (ATP) and carbon dioxide. 4 of the key components in the TCA cycle were detected, of these both isocitrate and malate increased while fumarate and succinate decreased. Results also showed that isocitrate increased the most (3 times) after fermentation. This is in line with studies that had shown that *B. subtilis* produced the enzyme aconitate hydratase which catalysed the stereo-specific isomerisation of citrate to isocitrate (Cox and Hanson 1968; Dingman et al. 1987). The large amount of isocitrate produced likely drove reactions forward to produce succinate and fumarate which are consumed to produce malate.

The intermediates of the TCA cycle are involved in numerous reactions that produced important compounds. Isocitrate are broken down by the action of isocitrate dehydrogenase into 2-oxoglutarate which can then be converted into the non-essential amino acid, glutamate. Glutamate are in turn converted into ornithine via acetylornithine deacetylase which is part of the urea cycle. Critically, ornithine cyclodeaminase catalyses the conversion of ornithine to proline which is an essential amino acid (arginine and proline metabolism pathway). Glutamate, ornithine and proline were all upregulated after fermentation which strengthens the hypothesis that isocitrate were produced in excess. 2-oxoglutarate can also be converted into lysine through the lysine biosynthesis pathway.

From the pathway analysis, aspartate played a vital role in the synthesis of numerous important compounds. Firstly, in the lysine biosynthesis pathway, aspartate is converted into lysine through catalysis by diaminopimelate decarboxylase. Aspartate can also be directly converted into the TCA cycle intermediate, fumarate by aspartase (alanine, aspartate and glutamate metabolism pathway). Next, aspartate is also directly converted into asparagine, a non-essential amino acid by asparagine synthetase (alanine, aspartate and glutamate metabolism pathway). Aspartate is also involved in the synthesis of the essential amino acid, methionine which had been linked with optimising the immune function of the human intestines (cysteine and methionine metabolism pathway)(Ruth and Field 2013). Lastly, aspartate can be converted to threonine through threonine synthase (glycine, threonine and serine metabolism pathway). Threonine is also produced from the reversible reaction of glycine through catalysis by threonine aldolase (glycine, serine, threonine metabolism pathway). Glycine, another essential amino acid is in turn produced from the reversible reaction of serine through catalysis by glycine hydroxymethyltransferase (glycine, serine, threonine metabolism pathway). In addition, serine can also be converted to cysteine through cysteine synthase (cysteine and methionine metabolism pathway).

It was also observed that all the amino acids detected were glucogenic amino acids which means that they can be converted into glucose through gluconeogenesis. This could explain why although all the amino acids were up regulated after fermentation, the overall amount detected after fermentation were much lesser compared to fatty acids.

The metabolic pathways for the biosynthesis of fatty acids involved the conversion of acetyl-coA to malonyl-coA through acetyl-coA carboxylase which were then converted to the saturated fatty acids, palmitate and stearate through the fatty acid biosynthesis pathway. Palmitate and stearate then underwent elongation and unsaturation process to yield oleate and linoleate (biosynthesis of unsaturated fatty acids pathway). Both palmitate and stearate were down regulated while oleate and linoleate were up regulated. This suggested that a larger proportion of the saturated fatty acids were converted into unsaturated fatty acids. The reduction in saturated fatty acids are desirable as it is well known that consumption of high amount of saturated fatty acids are associated with increased risk of coronary heart diseases (Zong et al. 2016).

Overall, from the metabolic pathway analysis (Fig. 6), it can be summarized that the amount of various carbohydrates in okara decreased. This suggested that carbohydrates were consumed and utilised by *B. subtilis* WX-17 through glycolysis, to produce energy for its metabolism and cellular process. Subsequently, this led to the increased antioxidant amount, amino acids and fatty acids in fermented okara.

In conclusion, with the world’s population predicted to reach 9 billion by 2050, food security is becoming a rising global issue (Godfray et al. 2010). One strategy to combat these issues is the biovalorisation of food waste. With the growing popularity of soy-based products, the amount of okara produced are increasing rapidly, approximately 14 million tonnes globally every year. This work showed the possibility of reintroducing okara, a food waste back into the food chain through biovalorisation by fermentation. As the microorganism used is food grade, this study also presents the potential application of fermented okara into human diets in various forms. The study revealed that fermentation of okara using food grade *B. subtilis* WX-17 enhanced its nutrient profile. GC–MS and metabolic pathway analysis showed that both amino acids and fatty acids production increased after fermentation due to the release of hydrolases by *B. subtilis* WX-17 to break down complex macromolecules into simpler molecules which are easier to digest. Furthermore, antioxidant analysis also showed that post fermentation, fermented okara displayed higher antioxidant activities which indicated a higher phenolic content. Future work will include characterising the enzymes produced by *B. subtilis* WX-17, such as nattokinase, as well as other important components such as the valuable vitamin K2 MK7. In addition, downstream processing to extract the bioactive compounds from fermented okara will be carried out.

## Abbreviations

*B. subtilis*: *Bacillus subtilis*
GC–MS: gas chromatography-mass spectrometry
TCA cycle: tricarboxylic acid cycle
DPPH: 1-1,-diphenyl-2-picryl-hydrazil
CVD: cardiovascular disease
SSF: solid state fermentation
SLF: submerged liquid fermentation
MSTFA: *N*-methyl-*N*-(trimethylsilyl) trifluoroacetamide
TMCS: trimethylchlorosilane
DMF: dimethylformamide
DNA: deoxyribonucleic acid
PCR: polymerase chain reaction
IS: internal standard
BF3-methanol: boron trifluoride methanol
PLS-DA: partial least squares discriminant analysis
RNA: ribonucleic acid
LDL: low density lipoprotein
KEGG: Kyoto Encyclopaedia of Genes and Genomes
G6P: glucose 6-phosphate
PEP: phosphoenolpyruvate
UDP: uridine diphosphate
ATP: adenosine triphosphate
